# Involvement of Centrally Projecting Edinger–Westphal Nucleus Neuropeptides in Actions of Addictive Drugs

**DOI:** 10.3390/brainsci10020067

**Published:** 2020-01-26

**Authors:** Alfredo Zuniga, Andrey E Ryabinin

**Affiliations:** Department of Behavioral Neuroscience, Oregon Health & Science University, 3181 SW Sam Jackson Park Road, Portland, OR 97219, USA; zuniga@ohsu.edu

**Keywords:** Edinger–Westphal nucleus, ethanol, addiction, neurocircuitry

## Abstract

The centrally-projecting Edinger–Westphal nucleus (EWcp) is a brain region distinct from the preganglionic Edinger–Westphal nucleus (EWpg). In contrast to the EWpg, the EWcp does not send projections to the ciliary ganglion and appears not to regulate oculomotor function. Instead, evidence is accumulating that the EWcp is extremely sensitive to alcohol and several other drugs of abuse. Studies using surgical, genetic knockout, and shRNA approaches further implicate the EWcp in the regulation of alcohol sensitivity and self-administration. The EWcp is also known as the site of preferential expression of urocortin 1, a peptide of the corticotropin-releasing factor family. However, neuroanatomical data indicate that the EWcp is not a monotypic brain region and consists of several distinct subpopulations of neurons. It is most likely that these subpopulations of the EWcp are differentially involved in the regulation of actions of addictive drugs. This review summarizes and analyzes the current literature of the EWcp’s involvement in actions of drugs of abuse in male and female subjects in light of the accumulating evidence of complexities of this brain region.

## 1. Introduction to the Centrally-Projecting Edinger-Westphal Nucleus

Since the mid-nineteenth century, the Edinger–Westphal nucleus (EW) has been traditionally described as a parasympathetic nucleus involved in oculomotor adaptation. Although many early studies contradicted this idea, including studies by Ramon Y Cajal [1], the projections to the ciliary ganglion from some of the neurons identified as EW neurons solidified this relatively simplistic interpretation of its structure and function (for a thorough review on this subject see [2]). Recent findings demonstrating that the EW projects centrally to other brain areas (see section below), and the lack of colocalization with markers of parasympathetic neurons and/or ciliary ganglion afferents [3,4,5,6] indicate that this region is far more complex than initially believed. Although several alternative nomenclatures of the EW have been proposed to reconcile the controversies, the field has come to an agreement to define subpopulations within this nucleus by their afferents [2]. Specifically, researchers distinguish at least two major subpopulations of neurons within the EW: the centrally projecting EW (EWcp) and the preganglionic EW (EWpg).

Consistent with its nomenclature, the EWcp projects to numerous regions of the CNS (see Projections of the EWcp, below). Neurochemically the EWcp can be identified by the expression of several reward-, stress- and energy expenditure-related neuropeptides, including Ucn1, cocaine and amphetamine-regulated transcript (CART), cholecystokinin (CCK), and substance P. Numerous studies have provided extensive evidence for the involvement of these neuropeptides, as well as the EWcp specifically in the actions of alcohol and other drugs of abuse, which are discussed below. 

## 2. Neuromodulators Produced in the EWcp

**Figure 1:**
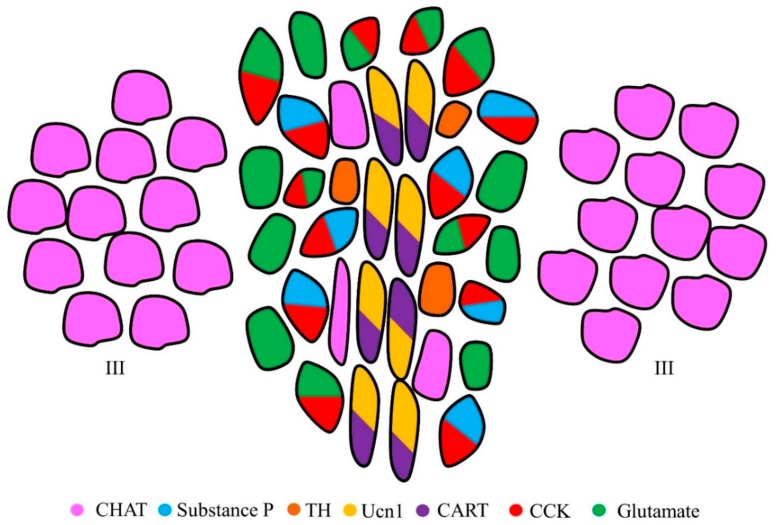
Illustration of the neuropeptides and neurotransmitters known to be expressed in the EWcp. This coronal illustration of the EWcp demonstrates that numerous neuropeptides are expressed within the same cells. The EWcp sits between CHAT-expressing cells of the oculomotor nucleus (III). EWcp neurons can be distinguished from the EWpg neurons based on their lack of CHAT expression. The few CHAT-positive neurons within the EWcp most likely represent the EWpg. EWpg, centrally-projecting Edinger–Westphal nucleus; CHAT, choline acetyltransferase; TH, tyrosine hydroxylase; Ucn1, urocortin 1; CART, cocaine and amphetamine-regulated transcript; CCK, cholecystokinin.

As mentioned above, the EWcp expresses a number of neuropeptides, including Ucn1, CART, CCK, and substance P, among others. As with other nuclei rich in neuropeptides, such as the paraventricular nucleus of the hypothalamus (PVN) [7], some of these neuropeptides are co-expressed within the same cells in the EWcp. Thus, it has become possible to classify and separate sub-populations of neurons within the EWcp based on the neuropeptides that they express (Figure 1). Indeed, Ucn1 and CART have been shown to be co-localized in a subset of EWcp neurons [8,9]. This population of neurons also co-expresses the less-studied peptide Nesfatin-1 [10]. A separate population of neurons within the EWcp has been shown to co-express CCK and substance P [11]. The EWcp has also been shown to contain a small population of TH-positive neurons, that normally do not colocalize with Ucn1 [12]. Based on their distinct distributions, it appears that CCK-, Ucn1- and TH-positive cells are distinct, and the three markers are not co-expressed in the same neurons. On the other hand, one report indicates that under certain pathological conditions Ucn1 and TH can be co-expressed in the human embryonic EWcp [13]. More recently, fluorescent in-situ hybridization (FISH) demonstrated that almost all CCK cells also express the gene Slc17a6 [14], encoding the vesicular glutamate transporter 2 (Vglut2), strongly suggesting that they are also glutamatergic [15,16]. Although almost all CCK-positive cells in the EWcp appear to express Slc17a6, Zhang and colleagues found that there were a number of Slc17a6-positive cells that did not express CCK [14]. To date it is not known what other neuropeptides these glutamatergic neurons may express, although preliminary data from the Ryabinin lab indicates that adult Vglut2 neurons in the EWcp do not express Ucn1. Currently it is also not definitively known if neurons that express CCK and Slc17a6 co-express substance P. However, this co-expression appears likely given the extent of CCK and substance P colocalization it the EWcp. Future immunohistochemical assays for substance P, CCK and Vlgut2 will provide further insight into these Vglut2-positive neurons. In addition, the EWcp is also known to be enriched in the stress-related peptide PACAP [17]. Currently it is not known with what other neuromodulators this peptide is co-expressed. The neuropeptides expressed in the EWcp have all been shown to be associated with sensitivity to drugs of abuse and have also been shown to regulate the intake and preference of a number of drugs. The specific roles of these neuropeptides are described in further detail in later chapters of this review.

## 3. Sensitivity of the EWcp to Alcohol and Other Drugs of Abuse

### 3.1. Sensitivity of Undefined Populations of the EWcp

To date, numerous studies have shown that the EWcp is highly sensitive to ethanol (EtOH) administration. Using the immediate early gene (IEG) c-Fos as a marker of neuronal activity, the EWcp has been repeatedly shown to be activated following experimenter-administered EtOH as well as voluntary drinking [12,18,19,20]. In rats, the only brain region activated following a limited access period for non-alcoholic beer that was supplemented with EtOH was the EWcp [21]. Rats given access to non-supplemented non-alcoholic beer, as well as saccharin or water, did not show these increases in c-Fos, suggesting that the activation of the EWcp was specific to the EtOH. In agreement with this finding, the EWcp was also the only brain region in the rat brain showing c-Fos induction following operant self-administration of EtOH or saccharin-supplemented EtOH [22]. Conversely, the EWcp did not show activation following self-administration of water or saccharin. In mice, increased c-Fos expression in the EWcp has been observed following limited-access to sucrose-sweetened EtOH, as well as EtOH alone, but not following sucrose, saccharin, or water [23,24,25,26]. Importantly, the amount of EtOH consumed by mice in a limited-access paradigm was positively correlated with the number of c-Fos positive cells in the EWcp [27]. C-Fos mRNA expression levels have also been found to positively correlate with EtOH intake in longer access paradigms (24-h) [28]. Increased neuronal activity in the EWcp has also been observed in prairie voles, a monogamous rodent species known to show high levels of intake and preference. Indeed, an increase in c-Fos expression in the EWcp has been observed following a 2 h limited access period [29]. Additionally, increased levels of FosB were observed in the EWcp after seven days of 24 h access to EtOH in prairie voles [30,31] and mice [23,32]. 

In addition to EtOH, several other drugs have been shown to increase c-Fos expression in the EWcp. Increased c-Fos expression has been seen following exposure to either heroin [33] or morphine [34]. Interestingly, the cannabinoid receptor 1 (CB1) antagonist SR141716 has also been shown to increase c-Fos expression in the EWcp when administered alone. When given in combination with morphine however, SR141716 did not increase c-Fos expression, indicating that the opioid and cannabinoid systems may interact to modulate neuronal activity in the EWcp [34]. The EWcp has also been shown to be sensitive (as measured by increased c-Fos expression) to psychostimulants cocaine and methamphetamine [35] in both mice and rats but not to nicotine [36]. 

### 3.2. Sensitivity of Specific Subpopulations of EWcp

Not all of the studies above have specifically tested whether the neurons of the EWcp that are sensitive to these drugs express Ucn1, or whether the drug-induced expression of immediate early genes occurs in other neuronal populations of the EWcp. Early double-labeling experiments indicated that induction of c-Fos following experimenter-administered EtOH is strongly colocalized with Ucn1 neurons in inbred C57BL/6J mice, but is equally abundant in non-Ucn1 neurons of the EWcp in DBA/2J mice [37]. Since C57BL/6J, but not DBA/2J mice, consume substantial amounts of alcohol, double-labeling of Ucn1 and c-Fos following voluntary alcohol consumption has only been performed in the former strain of mice. These studies indicated that nearly all c-Fos-expressing cells following alcohol drinking in C57BL/6J mice, were Ucn1-positive [25]. Cocaine-induced c-Fos expression was observed almost exclusively in Ucn1 neurons and was not observed in TH-positive cells of the EWcp [35]. More recently, Giardino and colleagues demonstrated that Ucn1 mRNA expression levels are increased 24 h after animals have been exposed to an intermittent access paradigm in which they had 22 h access to EtOH every other day, suggesting that EWcp-Ucn1 neurons are also sensitive to long-term exposure to EtOH [28]. 

Given that CART is almost completely co-expressed with Ucn1 in the EWcp, it is likely that CART-positive neurons are also sensitive to drugs of abuse. In agreement with this idea, offspring of rat dams exposed to alcohol from gestation to weaning show a ~2-fold increase in the number of CART-immunoreactive cells in the EWcp, compared to those born by mothers not given alcohol access [38]. Interestingly, this difference in the number of CART-positive cells was only visible during weaning, as there were no significant differences during adulthood. In contrast to the evidence demonstrating sensitivity of Ucn1 and CART neurons of the EWcp, TH-positive neurons of the EWcp don’t show any c-Fos induction following administration of EtOH or cocaine [35]. The sensitivity of other EWcp subpopulations to alcohol or other addictive substances has not been yet evaluated. 

## 4. Involvement of the EWcp in the Regulation of Alcohol Self-Administration

### 4.1. Involvement of Undefined Populations of the EWcp

Studies indicate that the EWcp is not only sensitive to alcohol and other drugs of abuse, but is also involved in the regulation of self-administration of such drugs, or at least in regulating the sensitivity to alcohol and alcohol intake. Surgical manipulations of the EWcp have provided additional evidence for the involvement of the EWcp in the regulation of EtOH intake. When mice received electrolytic lesions of the EWcp, they consumed significantly less EtOH than sham-operated controls, and also showed a reduced preference for EtOH [39]. The decrease in intake and preference observed in EWcp-lesioned mice was accompanied by an attenuation of EtOH-induced hypothermia, suggesting that the EWcp may also be involved in the regulation of the physiological effects of EtOH. The effects of lesions on consummatory behavior is not completely specific to EtOH, as such lesions also decrease food and water consumption. Interestingly, despite these effects, lesions of the EWcp do not significantly affect body weight, suggesting that the EWcp contributes to the regulation of energy expenditure [40], an effect in agreement with the EWcp’s contribution to the regulation of alcohol-induced hyperthermia. 

### 4.2. Involvement of Specific Subpopulations of the EWcp

#### 4.2.1. Urocortin 1

Of the several neuropeptides that are expressed in the EWcp, Ucn1 has been studied most extensively regarding its role in the regulation of EtOH intake. Although Ucn1 is primarily expressed in the EWcp, it is also produced in the lateral superior olive, and in the supraoptic nucleus, although at much lower levels [6,41,42,43]. As a member of the corticotropin releasing factor (CRF) family of neuropeptides, Ucn1 binds to both CRF receptors (CRF1R, CRF2R) with greater affinity than CRF itself [42,44]. Some of the literature on the contribution of Ucn1 to the regulation of alcohol consumption has been reviewed previously [45,46,47]. Ucn1’s role in consummatory behaviors was first shown in studies in which systemic administration of the peptide had robust anorexic effects [48]. The role of EWcp Ucn1 neurons in the regulation of EtOH intake is supported by several different lines of work. First, various genetic studies have demonstrated that baseline Ucn1 expression levels are higher in rodent strains with high levels of EtOH intake and preference, compared to those that consume lower levels of EtOH [6]. Multiple studies demonstrated that when animals were selectively bred to drink high levels of EtOH, predisposition to high EtOH intake was accompanied by relatively higher basal levels of Ucn1 immunoreactivity in the EWcp [12,20,49]. Within mice, it is well known that the C57BL/6J strain drinks much more EtOH than DBA/2J mice, and these differences in EtOH intake have also been associated with significantly higher levels of Ucn1 mRNA, as well as a higher number of Ucn1-expressing neurons in the EWcp of C57BL/6J mice [50,51]. In light of these findings, and since Ucn1 is primarily expressed in the EWcp, several studies have utilized the Ucn1 KO line to assess how genetic deletion of Ucn1 alters EtOH intake and preference. Remarkably, Ucn1 KO mice drink lower levels of EtOH than their WT littermates only when they have long-term 24 h access to EtOH [28], and not during a binge-like paradigm [52]. Similarly, when mice were exposed to a 12-day procedure in which they had 24 h access to increasing concentrations of EtOH (10%, 20%, 40%), Ucn1-KO mice displayed significantly lower levels of intake and preference compared to WT mice. In contrast, when the concentration of EtOH was maintained at 10%, Ucn1 KO and WT mice displayed similar intake of alcohol, suggesting that Ucn1 is involved in the regulation of escalating intake of this addictive drug [28]. Additionally, Ucn1-KO mice do not develop EtOH-induced place preference, suggesting that Ucn1 is necessary for the formation of EtOH-cue associations [53]. Interestingly, mice lacking functional CRF2Rs also did not develop place preference [50], suggesting that Ucn1 binding with CRF2Rs facilitates the rewarding properties of EtOH. The interpretation of knockout studies can be complicated by potential developmental compensations. Therefore, it is important that in addition to genetic approaches, viral RNA interference has been used to alter Ucn1 expression in the EWcp of adult C57BL/6J mice. Using an shRNA mediated knockdown of Ucn1 expression in the EWcp, Giardino et al. demonstrated that decreased Ucn1 expression can blunt EtOH intake in an escalating long-term intermittent access paradigm, without affecting food or fluid intake [28]. The contribution of Ucn1 to the regulation of self-administration of other drugs of abuse has not been previously examined, but the contribution of CRF2R signaling to reinstatement of cocaine seeking suggests this possibility [54]. 

Another important insight into the contribution of EWcp and Ucn1 to alcohol self-administration was provided in studies which combined KO and electrolytic approaches. When the EWcp was lesioned in Ucn1 WT and KO mice, preference for EtOH was decreased only in WT mice, highlighting that Ucn1 expression in the EWcp is necessary for high EtOH preference [53]. In contrast, EtOH intake was decreased in both WT and KO animals that received an EWcp lesion. The latter finding suggests that other neuromodulators within the EWcp may also contribute to the regulation of EtOH intake. Although the contribution of these neuromodulators to alcohol and drug self-administration has been studied, the specific role of EWcp neurons expressing these peptides has not been yet examined. Several lines of evidence suggest that some of them could also be involved in regulating alcohol self-administration. 

#### 4.2.2. CART

CART is a neuropeptide richly expressed in both the central nervous system and the periphery [55,56,57]. As the name suggests, it was identified as a gene responsive to cocaine and amphetamine administration [58]. The role of CART in reward and addictive behaviors has been studied and reviewed extensively, particularly with regards to psychostimulants [59]. In addition to being sensitive to psychostimulant administration, CART may regulate the effects of psychostimulants, as well as their self-administration. However, this work primarily focused on the role of striatal CART, clearly indicating importance of CART in the nucleus accumbens in addiction-related behaviors [60,61]

An initial study investigating CART KO mice indicated that CART may be also be involved in the regulation of EtOH intake, as KO mice had lower EtOH intake and preference in a 24 h 2-bottle-choice procedure when compared to their WT littermates [62]. Differences in CART expression have also been observed between mouse strains that show differing levels of preference for EtOH intake. C57BL/6J mice, a strain known to exhibit high levels of EtOH intake and preference, show remarkably higher levels of both CART mRNA and protein in the EWcp, when compared to a strain with lower EtOH intake and preference (DBA/2J) [50]. Given the almost uniform co-expression of Ucn1 and CART in the EWcp, and the similar differences in Ucn1 expression between these two strains (see above), one is tempted to hypothesize that these two neuropeptides have related functions in the regulation of EtOH intake. Future studies assessing the involvement of CART in EtOH-seeking behaviors will provide much needed insight into this idea. 

#### 4.2.3. Cholecystokinin

CCK is a peptide expressed in the gut and the nervous system, including the EWcp [50,63,64]. Studies in the early 1970s demonstrated that CCK dose-dependently decreased food intake in food-deprived rats [65]. Its role as a satiety endocrine neuropeptide has been further supported through a number of studies since then (for review see [66]). CCK has been shown to be colocalized with dopamine (DA) in 80%–90% of neurons in the VTA, and terminals containing both CCK and DA have been detected in the NAcc and the CeA [67,68]. It has been hypothesized that CCK modulates DA function by acting on CCKA and CCKB receptors in the NAcc, although this relationship is believed to vary depending on specific parts of the NAcc [69,70,71]. 

The potential role of CCK in drug seeking behaviors comes from studies involving amphetamine, cocaine, and EtOH. Indeed, activation of CCKB receptors in the NAcc decreases the break point for intravenous self-administration of amphetamine. Furthermore, both CCKA and CCKB receptors have been shown to be critical to amphetamine sensitization [72]. Specifically, CCKB receptors may be more involved in the acute effects of amphetamine, while CCKA receptors may modulate long-term effects of the drug [72]. CCKA receptors also appear to be involved in regulating sensitization to cocaine, as rats lacking the receptor develop less behavioral sensitization than their WT counterparts [73]. 

Systemic injections of CCK have also been shown to decrease intake of EtOH, but not water, in rats [74,75]. In a series of studies, Crespi and colleagues demonstrated that CCK-mediated decreases occur via the CCKA receptor [76,77]. However, whether any of these effects are mediated by EWcp CCK neurons is currently unknown.

#### 4.2.4. Substance P

SP is an 11-amino acid neuropeptide that belongs to the tachykinin family of neuropeptides [78]. Of the three receptors that exist within the tachykinin family, SP is known to preferentially bind to the neurokinin 1 receptor (NK1R) [79]. NK1Rs are expressed in a number of stress- and reward-related regions in the brain, including the CeA, BNST, paraventricular nucleus of the thalamus (PVT), the DRN, VTA, and the locus coeruleus (LC) [80]. SP expression has been observed in the rodent [81,82] and feline EWcp [83,84], suggesting that expression of the neuropeptide in this brain region has been conserved across species. In rodents, substance P and CCK are co-expressed in a sub-population of EWcp neurons that project to the PVT [81]. 

A number of studies have implicated the SP-NK1R system in alcohol-related behaviors. When compared to WT mice, NK1R-KO mice have lower EtOH intake levels and do not develop EtOH-CPP. Furthermore, mice lacking NK1R appear to be more sensitive to the effects of EtOH, as they require more time to regain the righting reflex after receiving an EtOH injection [85]. Pharmacological antagonism of the NK1R has also been shown to dose-dependently decrease EtOH intake [86]. This was not observed in NK1R-KO mice indicating that this effect is specific for the receptor. The involvement of the SP-NK1R system in EtOH-related behaviors has also been investigated and demonstrated in rats. Indeed, stress-induced reinstatement of EtOH seeking has also been prevented by a systemic injection of an NK1R antagonist given prior to the reinstatement session [87]. In alcohol-preferring rats, intra-CeA microinfusion of SP reduced responding for EtOH, but not sucrose in an operant SA procedure [88]. Interestingly, since it known that the EWcp projects to the CeA (a region that expresses NK1Rs), it is possible that the EWcp may be a source of SP onto the CeA. 

## 5. Projections of the EWcp

**Figure 2:**
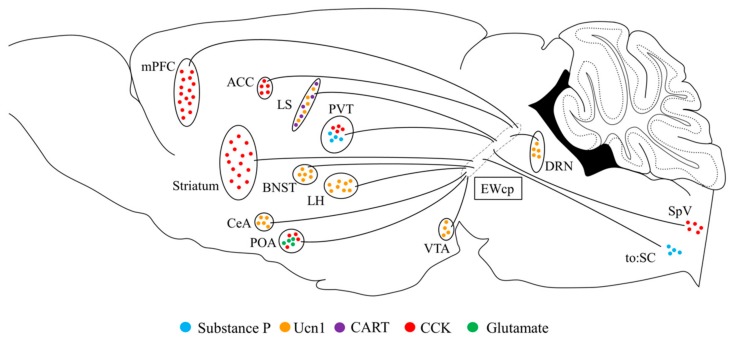
Schematic of central projections arising from the EWcp. Efferents originating in the EWcp project to a number of regions through the brain. These regions receive specific neuropeptidergic and neurotransmitter inputs from the EWcp. Filled circles denote known peptides and neurotransmitter known to be released in that area from the EWcp. Orange circles, Ucn1; purple circles, CART; blue circles, substance P; red circles, CCK; green circles, glutamate; ACC, anterior cingulate cortex; BNST, bed nucleus of the stria terminalis; CeA, central nucleus of the amygdala; DRN, dorsal raphe nucleus; EWcp, centrally-projecting Edinger–Westphal nucleus; LH, lateral hypothalamus; LS, later septum; mPFC, medial prefrontal cortex; POA, preoptic area; PVT, paraventricular nucleus of the thalamus; SC, spinal cord; SpV, trigeminal nucleus; VTA, ventral tegmental area.

A number of studies have added to our understanding of the neurocircuitry of the EWcp. In particular, these previous studies have made it possible to distinguish the projections of the EWcp based on the neuropeptides that are released in target areas (Figure 2). Tracing studies conducted in the 1980s demonstrated the existence of substance P and CCK projections from the EWcp to the spinal cord [63,84]. In addition, CCK-positive neurons in the EWcp project to the trigeminal nucleus [63]. Interestingly, these CCK- and substance-P-expressing neurons were shown to be sensitive to noxious stimuli [11]. Substance P and CCK afferents originating from the EWcp have also been detected in the paraventricular thalamic nucleus, where they have been hypothesized to regulate stress responses [81]. Most recently, EWcp CCK-positive cells were shown to project to the pre-optic area (POA) [14]. Using fluorescent in-situ hybridization, this study found that nearly all of the CCK-positive neurons they examined also expressed Slc17a6, indicating that they are also glutamatergic, and thus may also release glutamate in the POA.

The lateral septum (LS) is a major forebrain target of the EWcp, and as such considerable attention has been given to characterizing EWcp-LS projections [89]. CART- and Ucn1-positive fibers from the EWcp have been shown to densely innervate the LS [8]. Although both fiber terminals are detected throughout the lateral septum, it appears that Ucn1-positive fiber terminals tend to be located in the ventrolateral part of the LS, while those that are CART-positive appear to be more concentrated towards the medial sections of the LS. The level of EWcp-LS Ucn1 innervation appears to vary greatly by mouse strain, as D2 mice have more Ucn1 processes than B6 mice [90]. An analysis of B6D2 F2 offspring found a negative correlation between EtOH consumption and Ucn1 processes in the LS, indicating that EWcp-LS Ucn1 projections may influence EtOH intake. Additionally, evidence exists indicating that Ucn1-positive fibers in the LS are decreased following prolonged exposure to EtOH in both B6 and D2 mice [40]. Differences in Ucn1 fibers in the LS have also been found in rats selectively bred to drink EtOH, with those rats that prefer EtOH having higher levels of Ucn1 fibers in the LS than those that do not [20]. Although the exact effects of these differences in fiber density between species and strains is not yet fully understood it seems rather likely that EWcp-LS projections may be involved in the regulation of EtOH intake. 

Like the LS, the dorsal raphe nucleus (DRN) contains Ucn1-positive fibers originating in the EWcp that are sensitive to EtOH exposure. Indeed, following 7 days of EtOH exposure via IP injections, the density of Ucn1-positive fibers is decreased in the DRN [40]. Ucn1 fibers in the DRN have also been shown to be significantly decreased following electrolytic lesions of the EWcp [39], further confirming that the EWcp sends Ucn1 projections to the DRN. A thorough characterization of Ucn1-positive fibers, as well as the differences in Ucn1 fiber density between D2 and B6 mice was conducted by Weitemier and colleagues [6]. Interestingly, a number of regions with substantial Ucn1 innervation overlap with the findings obtained by tracing by Da Silva et al (discussed below), including the central nucleus of the amygdala (CeA), ventral tegmental area (VTA) and the bed nucleus of the stria terminalis (BNST), three regions which have been heavily implicated in the regulation of EtOH intake and preference. 

In addition to the studies listed above, a set of comprehensive tracing studies investigating the ascending and descending projections of the EWcp have been performed recently in rats [91,92]. Using a combination of anterograde tracing techniques, work from the Bittencourt laboratory has provided critical information regarding the neurocircuitry of the EWcp. Using a semi-quantitative comparative analyses, these studies classified the amount of EWcp-originating labeled fibers as being either “few”, “moderate”, or “many”. Within the prosencephalon, regions that displayed moderate levels of labeled fibers included the orbital cortex, the basolateral amygdala (BLA), and the anterior amygdaloid area. Many fibers were detected in the LS, the anterodorsal part of the BNST, and the ventral pallidum. Furthermore, the CeA and the oval part of the BNST displayed the densest amount of fibers. Several regions of the diencephalon showed moderate levels of fibers, including the paraventricular nucleus of the hypothalamus (PVN), as well as a number of regions within the thalamus. Only the lateral hypothalamic area and the reuniens thalamic nucleus displayed high amounts of fibers in the diencephalon. Within the brainstem, a moderate amount of fibers were detected in the ventrolateral and anterior sections of the periaqueductal gray, the substantia nigra parts compacta SNc, and the ventral and dorsal parts of the dorsal raphe nucleus (DRN). A more recent study also showed that CCK neurons of EWcp project to the medial prefrontal cortex (mPFC), anterior cingulate (ACC) and striatum [93].

## 6. Limitations and Future Directions

Early studies on afferent projections to the EWcp were constrained by the misunderstanding that they were analyzing afferents of the classical Edinger–Westphal nucleus (now known as the EWpg). More recent studies, recognizing that the anatomically-defined Edinger–Westphal nucleus in rodents is comprised primarily of the EWcp, showed presence of fibers from the VTA. These fibers extended into the vicinity of Ucn1 neurons of the mouse EWcp [12]. A depletion of these dopaminergic terminals did not prevent lipopolysaccharide-induced c-Fos induction in the EWcp [94]. A subsequent study indicated this projection to the EWcp is inhibitory, such that inhibition of activity in the VTA resulted in c-Fos induction in the EWcp [95]. A systematic assessment of retrograde tracing from the EWcp in rats identified afferent neurons in the subfornical area, paraventricular nucleus of hypothalamus, arcuate nucleus, lateral hypothalamus, zona inserta, posterior hypothalamus, medial vestibular nucleus and cerebellar interpositus nucleus [92]. Subsequent anterograde tracing experiments indicated that the lateral, paraventricular and posterior hypothalamic neurons extended fibers onto Ucn1 neurons. A recent study also identified an excitatory projection to the EWcp from the ventral hippocampus. It appears likely, that this projection was more specific to CCK neurons of the EWcp [93]. Despite the recent advances, the complexity of the EWcp calls for investigation of projections to specific neuronal subpopulations within the EWcp.

While the Ucn1 neurons of the EWcp are highly sensitive to drugs of abuse, there is also a substantial literature indicating that these neurons are sensitive to various environmental stressors [8,96,97,98]. More recent studies show a role of theEWcp in the regulation of attention and vigilance [93,99]. In contrast, studies in mice showed that alcohol-induced activation of the EWcp is not simply a response to an environmental stressor [100]. Moreover, lesions of the mouse EWcp do not result in overt changes in anxiety, suggesting that this brain region may not contribute significantly to the response to a stressor [39]. Since environmental stressors strongly affect responses to drugs of abuse and the development of addiction, whether there is a contribution of specific subpopulations of the EWcp to stress responsivity needs to be examined in greater detail.

There are well documented sex differences in sensitivity to drugs of abuse and propensity to addiction. Sex differences in Ucn1, CART and Nesfatin-1 levels in the EWcp of human suicide victims have been described [10]. Ucn1 levels in the rat EWcp have also been shown to differ between males and females, and have also been shown to vary depending on the estrous cycle [101]. This sex-dependent regulation could be due to the presence of estrogen receptor beta on Ucn1 neurons [102]. As an alternative explanation, the EWcp appears to be enriched in paternally-expressed genes [50]. Some of the studies on the role of the EWcp in response to drugs of abuse have included males and females in their analysis. C-Fos induction in the EWcp of male and female mice appeared to be equally sensitive to alcohol [37]. The majority of alcohol-related phenotypes was also not dependent on sex in Ucn1 KO mice. However, alcohol preference (but not consumption) in a limited access procedure was higher in Ucn1 knockout males versus females, and the locomotor-suppressive effects of EtOH was greater in Ucn1 knockout females versus males [28]. Therefore, sex differences in the EWcp’s contribution to addiction-related phenotypes should be studied in greater detail.

Finally, early studies of the EWcp described substantial differences in the structure and neurochemical composition of this brain region between different strains of rodents [37,51]. There is also a substantial difference in neuronal subpopulations of the EWcp between different species of animals [2,103]. Therefore, it seems likely that the composition and sensitivity to various factors will be different between humans and rodents. Therefore, the activity of the EWcp needs to be studied in humans. fMRI studies in humans show that differences in activity of the Edinger–Westphal nucleus correlate with differential responses to sad faces suggesting its involvement in emotional responses, however the resolution of these studies are beyond the ability to recognize the EWcp [104]. Postmortem studies show elevated Ucn1 levels in the EWcp of suicide victims [105]. These few studies indicate that the human EWcp is involved in emotional responses. A thorough analysis of subpopulations of EWcp neurons in humans following exposure to drugs of abuse has not yet been conducted, and merits consideration in the future.

## 7. Conclusions

Accumulating evidence indicates that the EWcp is a complex brain region consisting of several populations of neurons with distinct neurochemistry and function. IEG studies examining c-Fos expression following experimenter administered-EtOH, as well as oral intake of EtOH clearly demonstrate that Ucn1- and CART-positive neurons in the EWcp are highly sensitive to EtOH. In addition, studies that have focused on other drugs of abuse have shown that this same population of neurons is also sensitive to other drugs of abuse, such as cocaine and methamphetamine. In contrast to these populations of neurons, TH positive cells appear to not be activated by drugs of abuse, as demonstrated by lack of c-Fos expression in TH-positive neurons in the EWcp following EtOH and stimulant administration. Early studies utilizing electrolytic lesions of the EWcp demonstrated that this region can regulate EtOH intake. More recent work using more specific techniques has confirmed that Ucn1 within the EWcp may regulate EtOH consumption. Given the diverse populations of neuropeptides expressed in the EWcp, future work characterizing the involvement of other populations will be critical for our understanding of the EWcp in the involvement of EtOH intake and drug-seeking behaviors.
